# Frequent epigenetic inactivation of Wnt antagonist genes in breast cancer

**DOI:** 10.1038/sj.bjc.6604259

**Published:** 2008-02-19

**Authors:** H Suzuki, M Toyota, H Caraway, E Gabrielson, T Ohmura, T Fujikane, N Nishikawa, Y Sogabe, M Nojima, T Sonoda, M Mori, K Hirata, K Imai, Y Shinomura, S B Baylin, T Tokino

**Affiliations:** 1 First Department of Internal Medicine, Sapporo Medical University, Sapporo, Japan; 2 Department of Molecular Biology, Cancer Research Institute, Sapporo Medical University, Sapporo, Japan; 3 PRESTO, Japan Science and Technology Corporation, Kawaguchi, Japan; 4 The Sidney Kimmel Comprehensive Cancer Center at Johns Hopkins, Johns Hopkins University School of Medicine, Baltimore, MD, USA; 5 Department of Pathology, Johns Hopkins University School of Medicine, Baltimore, MD, USA; 6 First Department of Surgery, Sapporo Medical University, Sapporo, Japan; 7 Department of Public Health, Sapporo Medical University, Sapporo, Japan; 8 President, Sapporo Medical University, Sapporo, Japan

**Keywords:** Wnt, SFRP, DKK1, methylation, breast cancer

## Abstract

Although mutation of *APC* or *CTNNB1* (*β*-catenin) is rare in breast cancer, activation of Wnt signalling is nonetheless thought to play an important role in breast tumorigenesis, and epigenetic silencing of Wnt antagonist genes, including the secreted frizzled-related protein (SFRP) and Dickkopf (DKK) families, has been observed in various tumours. In breast cancer, frequent methylation and silencing of *SFRP1* was recently documented; however, altered expression of other Wnt antagonist genes is largely unknown. In the present study, we found frequent methylation of *SFRP* family genes in breast cancer cell lines (*SFRP1*, 7 out of 11, 64%; *SFRP2*, 11 out of 11, 100%; *SFRP5*, 10 out of 11, 91%) and primary breast tumours (*SFRP1*, 31 out of 78, 40%; *SFRP2*, 60 out of 78, 77%; *SFRP5*, 55 out of 78, 71%). We also observed methylation of *DKK1*, although less frequently, in cell lines (3 out of 11, 27%) and primary tumours (15 out of 78, 19%). Breast cancer cell lines express various Wnt ligands, and overexpression of SFRPs inhibited cancer cell growth. In addition, overexpression of a *β*-catenin mutant and depletion of SFRP1 using small interfering RNA synergistically upregulated transcriptional activity of T-cell factor/lymphocyte enhancer factor. Our results confirm the frequent methylation and silencing of Wnt antagonist genes in breast cancer, and suggest that their loss of function contributes to activation of Wnt signalling in breast carcinogenesis.

Wnt ligands are secreted proteins that bind to transmembrane receptors in the Frizzled (Fz) family. During normal developmental processes, the resultant Wnt signalling plays essential roles in the regulation of cell proliferation, patterning and fate determination (Cadigan and Nusse, 1997). The binding of Wnt to Fz leads to dephosphorylation and stabilisation of *β*-catenin, enabling it to be translocated into the nucleus, where it interacts with members of the T-cell factor/lymphocyte enhancer factor (TCF/LEF) family of transcription factors to stimulate the expression of target genes. This signalling pathway is strongly implicated in tumorigenesis; indeed, the first mammalian Wnt isoform was identified based on its ability to promote mouse mammary tumorigenesis (Polakis, 2000). In addition, aberrant nuclear and cytoplasmic localisation of *β*-catenin is frequently observed in human breast cancer (Lin *et al*, 2000; Ryo *et al*, 2001; Chung *et al*, 2004). In contrast to colorectal cancer (CRC), however, mutation of *APC*, *AXIN* or *CTNNB1* (*β*-catenin) is rare in breast cancer, indicating that other mechanisms are responsible for the activation of *β*-catenin. These mechanisms could include increased expression of Wnt ligand (Huguet *et al*, 1994; Dale *et al*, 1996; Bui *et al*, 1997) and/or the loss of Wnt antagonists.

Several classes of secreted Wnt antagonists are known, including the Cerberus, Wnt inhibitory factor 1, secreted frizzled-related protein (SFRP) and the Dickkopf (DKK) families (Kawano and Kypta, 2003). The SFRP family is comprised of five secreted glycoproteins identified as putative inhibitors of the Wnt signalling pathway (Jones and Jomary, 2002). Secreted frizzled-related proteins contain an N-terminal domain homologous to the cysteine-rich domain (CRD) of Fz and a C-terminal domain with some homology to netrin. This enables SFRPs to downregulate Wnt signalling by competing with Fz for Wnt binding via its CRD domain or by binding directly to Fz (Jones and Jomary, 2002). Vertebrates express four DKK proteins and a unique DKK3-related protein called Soggy (Niehrs, 2006). Dickkopf-1 binds to low-density lipoprotein receptor-related protein 5/6 (LRP5/6) and another transmembrane protein, Kremen, to selectively inhibit the canonical Wnt pathway by preventing Wnt and Fz from forming a ternary complex with LRP5/6 (Niehrs, 2006).

Epigenetic silencing of tumour suppressor and tumour-related genes in association with promoter CpG island hypermethylation is a frequent event that has been seen in virtually every tumour type (Ting *et al*, 2006). When we screened for epigenetically silenced genes in CRC, we found that multiple members of the *SFRP* family are concurrently methylated at high frequencies among cultured CRC cells and primary CRC specimens (Suzuki *et al*, 2002). We also found evidence that loss of SFRP function contributes to the activation of Wnt signalling in CRC cells (Suzuki *et al*, 2004). That *SFRP1* is located in a chromosomal region that is frequently deleted in breast cancer (8p12–p11.1) suggests that SFRP1 may also play a tumour suppressor role during mammary tumorigenesis (Ugolini *et al*, 1999, 2001; Armes *et al*, 2004). Consistent with that idea, we have previously found that *SFRP1* is methylated in several breast cancer cell lines (Suzuki *et al*, 2002), and two other groups recently reported frequent *SFRP1* methylation in both primary and cultured breast cancer cells (Lo *et al*, 2006; Veeck *et al*, 2006). To date, epigenetic silencing of *SFRP1* has been identified in a variety of malignancies, including cancers of the bladder (Stoehr *et al*, 2004), prostate (Lodygin *et al*, 2005), lung (Fukui *et al*, 2005) and breast, although abnormalities involving other *SFRP* family genes are largely unexplored.

We previously showed that *SFRP1*, *SFRP2* and *SFRP5* are frequently inactivated in CRC and gastric cancer (GC) (Suzuki *et al*, 2002; Nojima *et al*, 2007), and that SFRPs suppress constitutive Wnt signalling when overexpressed in CRC and GC cells. Similarly, Bafico *et al* (2004) reported that constitutive Wnt signalling could be suppressed in breast cancer cells by SFRP1 and DKK1. *DKK1* also has been shown to be a target of methylation-associated silencing in CRC cells (Aguilera *et al*, 2006). In the present study, we attempted to characterise the epigenetic abnormalities of Wnt antagonist genes in breast cancer and to determine whether *SFRP* genes function as tumour suppressors in the breast.

## MATERIALS AND METHODS

### Cell lines and tissue specimens

Six breast cancer cell lines (MDA-MB-231, MDA-MB-435S, MDA-MB-436, MDA-MB-468, MDA-MB-157, MDA-MB-453) were purchased from the American Type Culture Collection (Manassas, VA, USA), and five (MCF-7, T-47D, SK-BR-3, MDA-MB-361 and ZR-75-1) were purchased from Dainippon Sumitomo Pharma (Osaka, Japan). The two CRC cell lines (RKO and DKO2) used were described previously (Toyota *et al*, 2003). All of the cell lines were cultured in the appropriate medium. To analyse restoration of *SFRP* gene expression, cells were treated with 2 *μ*M 5-aza-2′-deoxycytidine (DAC) (Sigma, St Louis, MO, USA) for 72 h, replacing the drug and medium every 24 h. A total of 38 breast tumour specimens were obtained from Department of Pathology at Johns Hopkins Medicine (Baltimore, MD, USA). In addition, 78 primary breast tumour specimens from 78 Japanese patients were obtained from the First Department of Surgery, Sapporo Medical University. Informed consent was obtained from all patients before collection of the specimens. Genomic DNA was extracted using the standard phenol–chloroform procedure. Total RNA was extracted by using Trizol reagent (Invitrogen, Carlsbad, CA, USA) and then treated with a DNA-free kit (Ambion, Austin, TX, USA). Total RNA from normal breast tissue from a healthy individual was purchased from BioChain (Hayward, CA, USA).

### Reverse transcriptase-PCR

Single-stranded cDNA was prepared using SuperScript III reverse transcriptase (RT) (Invitrogen). The integrity of the cDNA was confirmed by amplifying glyceraldehyde-3-phosphate dehydrogenase (*GAPDH*). PCR was run in a 50-*μ*l volume containing 100 ng of cDNA, 1 × Ex Taq Buffer (TaKaRa, Otsu, Japan), 0.3 mM dNTP, 0.25 *μ*M each primer and 1 U of TaKaRa Ex Taq Hot Start Version (TaKaRa). The PCR protocol entailed 5 min at 95°C; 35 cycles of 1 min at 95°C, 1 min at 55°C and 1 min at 72°C; and a 7-min final extension at 72°C. Primer sequences for RT-PCR analysis are listed in Table 1. Real-time RT-PCR was carried out using SYBR Green sequence detection reagents (Applied Biosystems, Foster City, CA, USA) in a 50-*μ*l volume containing l00 ng of cDNA, 25 *μ*l of SYBR Green PCR Master Mix (Applied Biosystems) and 0.2 *μ*M each primer. The PCR protocol entailed 5 min at 95°C and 40 cycles of 30 s at 95°C and 1 min at 60°C. Fluorescent signals were detected using an ABI 7000 Prism 7000 (Applied Biosystems), and the accumulation of PCR product was measured in real time as the increase in SYBR green fluorescence. Data were analysed using ABI Prism 7000 SDS Software (Applied Biosystems). Standard curves relating initial template copy number to fluorescence and amplification cycle were generated using the amplified PCR product as a template, and were used to calculate mRNA copy number in each sample. Ratios of the intensities of the target genes and *GAPDH* signals were used as a relative measure of the expression level of target genes. Primer sequences for real-time RT-PCR are listed in Table 1.

### Methylation analysis

Bisulphite treatment of genomic DNA was performed as described previously (Suzuki *et al*, 2002). Methylation was then analysed using methylation-specific PCR (MSP) and bisulphite sequencing. PCR was run in a 25-*μ*l volume containing 50 ng bisulphite-treated DNA, 1 × MSP buffer (67 mM Tris-HCl (pH 8.8), 16.6 mM (NH_4_)_2_SO_4_, 6.7 mM MgCl_2_ and 10 mM 2-mercaptoethanol), 1.25 mM dNTP, 0.4 *μ*M each primer and 0.5 U of JumpStart REDTaq DNA Polymerase (Sigma). The PCR protocol for MSP entailed 5 min at 95°C; 35 cycles of 30 s at 95°C, 30 s at 60°C and 30 s at 72°C; and a 7-min final extension at 72°C. The PCR protocol for bisulphite sequencing entailed 5 min at 95°C; 35 cycles of 1 min at 95°C, 1 min at 60°C and 1 min at 72°C; and a 7-min final extension at 72°C. Amplified bisulphite-sequencing PCR products were cloned into pCR2.1-TOPO vector (Invitrogen), and 10 clones from each sample were sequenced. Primer sequences are listed in Table 1.

### Immunofluorescence microscopy

Cells cultured on chamber slides were washed with PBS and fixed in 4% paraformaldehyde, after which they were incubated with anti-*β*-catenin monoclonal antibody (BD Transduction Laboratories, San Diego, CA, USA) and stained with anti-mouse IgG conjugated with Alexa Fluor 488 (Invitrogen). Cells were then examined using an FV300-IX71 confocal laser scanning microscope (Olympus, Tokyo, Japan).

### Colony formation assays

Cells (2 × 10^6^ cells) were transfected with 5 *μ*g of one of the pcDNA3.1/His-SFRP vectors or with empty vector using the Cell Line Nucleofector kit V (Amaxa, Cologne, Germany) and a Nucleofector I electroporation device (Amaxa), according to the manufacture's instructions. The SFRP vectors were prepared as described previously (Suzuki *et al*, 2004), and an empty vector, pcDNA3.1/HisA (Invitrogen), was used as a control. Cells were plated on 60-mm culture dishes and selected for 14 days with 0.6 mg ml^−1^ G418, after which colonies were stained with Giemsa and counted using National Institute of Health IMAGE software.

### Flow cytometry

Fluorescence-activated cell sorting (FACS) analysis was carried out as described previously (Nojima *et al*, 2007). Briefly, 2 × 10^6^ cells were transfected with one of the SFRP vectors or with an empty vector as described above. Forty-eight hours after transfection, the cells were harvested, fixed with methanol, rehydrated with PBS, treated with 2 *μ*g ml^−1^ RNase for 30 min at 37°C, stained in propidium iodide solution (50 *μ*g ml^−1^) and analysed using a FACSCalibur instrument (Becton Dickinson, San Jose, CA, USA).

### Small interfering RNA-mediated knockdown of SFRP1

For small interfering RNA (siRNA)-mediated knockdown of *SFRP1*, three different oligonucleotide dsRNAs against SFRP1 (siSFRP1) were generated by Dharmacon (Lafayette, CO, USA), after which a mixture of the three was used for transfection. The sequences of the siRNA oligonucleotides are listed in Table 1. A negative control siRNA, siCONTROL, was also purchased from Dharmacon. Cells (2 × 10^6^ cells) were transfected with 1.5 *μ*g of siSFRP1 or siCONTROL using the Cell Line Nucleofector kit V (Amaxa) and a Nucleofector I electroporation device (Amaxa), according to the manufacturer's instructions. Total RNA was extracted 48 h after transfection, and *SFRP1* expression was analysed by RT-PCR.

### Cell viability assay

Proliferation of siRNA transfectants was analysed by measuring the uptake of tritium thymidine in 3-(4,5-dimethylthiazol-2-yl)-2,5-diphenyltetrazolium bromide (MTT) assays. Transfected cells were seeded into 96-well plates to a density of 5 × 10^3^ cells per well. After incubation for 48 h, MTT assays were carried out using a Cell Counting kit-8 (Dojindo, Tokyo, Japan), according to the manufacturer's instructions. The colorimetric read-out in this assay reflects the number of metabolically active mitochondria, and thus viable cells, in a given well.

### Analysis of TCF/LEF-mediated transcription

An expression vector encoding a mutant form of *β*-catenin, pcDNA3.1-*β*-cateninΔ45, was prepared as described previously (Suzuki *et al*, 2004). For the combination analysis of mutant *β*-catenin and siSFRP1, MDA-MB-436 cells were cotransfected with 5 *μ*g of pcDNA3.1-*β*-cateninΔ45 or empty vector plus 1.5 *μ*g of siSFRP1 or siCONTROL using the Cell Line Nucleofector kit V (Amaxa). RNA was prepared 48 h after transfection, and RT-PCR was performed as described above. For TCF/LEF-responsive reporter assays, MDA-MB-436 cells were transfected with 100 ng of pGL3-OT (a TCF/LEF-responsive luciferase reporter plasmid) or pGL3-OF (a negative control plasmid), 100 ng of pcDNA3.1-*β*-cateninΔ45 or empty vector, 1 ng of pRL-CMV and 100 nM of siSFRP1 or siCONTROL using Lipofectamine 2000 (Invitrogen). Firefly and *Renilla* luciferase activities were measured 48 h after transfection using a Dual-Luciferase Reporter Assay System (Promega, Madison, WI, USA) and a Lumat LB 9507 luminometer (Berthold Technologies, Bad Wildbad, Germany).

### Statistical analysis

Statistical analyses were carried out using SPSS software (version 11.0; SPSS Inc., Chicago, IL, USA). Mann–Whitney's *U*-test and Fisher's exact test (two-sided) were used to evaluate the association between methylation of Wnt antagonist genes and clinicopathological features. Values of *P*<0.05 were considered significant.

## RESULTS

### Analysis of Wnt antagonist gene expression in breast cancer cell lines

**Figure 1:**
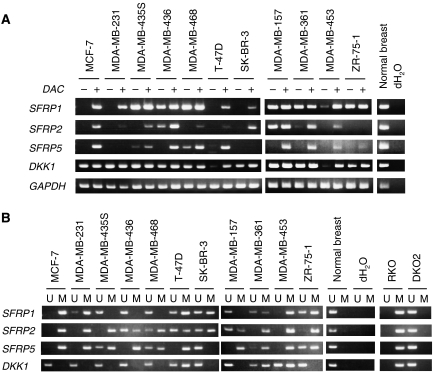
Analysis of the expression and methylation of Wnt antagonist genes in breast cancer cell lines. (**A**) RT-PCR analysis of *SFRP1*, *SFRP2*, *SFRP5* and *DKK1* expression in the indicated breast cancer cell lines, with and without DAC treatment, and in a normal breast tissue. Expression of *GAPDH* was assessed in all samples to ensure the cDNA quality; dH_2_O indicates no RNA added. (**B**) MSP analysis of the indicated breast cancer cell lines and normal breast tissue. A methylated CRC cell line (RKO) and another CRC cell line in which DNMT1 and DNMT3B were genetically disrupted (double knockout; DKO2), respectively, served as positive and negative controls of methylation. Bands in the ‘M’ lanes are PCR products obtained with methylation-specific primers; those in the ‘U’ lanes are products obtained with unmethylated-specific primers; dH_2_O indicates no DNA added.

We previously reported that three of the five *SFRP* genes (*SFRP1*, *SFRP2* and *SFRP5*) were frequently methylated and silenced in CRC and GC cells (Suzuki *et al*, 2002; Nojima *et al*, 2007). For that reason, in the present study we initially evaluated the expression status of *SFRP1*, *SFRP2* and *SFRP5* in a panel of breast cancer cell lines. We found that SFRP1 mRNA was completely absent in 4 of the 11 cell lines tested (MCF-7, MDA-MB-231, T-47D and SK-BR-3) and was downregulated in one cell line (MDA-MB-453) (Figure 1A). Treating the cells with the DNA methyltransferase (DNMT) inhibitor DAC rapidly restored its expression (Figure 1A).

*SFRP2* expression was absent in eight cell lines (MCF-7, MDA-MB-231, MDA-MB-435S, MDA-MB-468, T-47D, SK-BR-3, MDA-MB-453 and ZR-75-1) and downregulated in two (MDA-MB-436 and MDA-MB-361) (Figure 1A), while *SFRP5* expression was absent in nine cell lines (MCF-7, MDA-MB-231, MDA-MB-436, T-47D, SK-BR-3, MDA-MB-157, MDA-MB-361, MDA-MB-453 and ZR-75-1) and downregulated in two (MDA-MB-435S and MDA-MB-468) (Figure 1A). 5-Aza-2′-deoxycytidine treatment restored mRNA expression in the majority of the cells in which *SFRP2* and/or *SFRP5* were downregulated (Figure 1A).

As *DKK1* was recently shown to be epigenetically silenced in CRC, we also analysed expression of *DKK1* in the breast cancer cells. We found that DKK1 mRNA was significantly downregulated in two cell lines (T-47D and MDA-MB-453), and the expression was restored by DAC treatment (Figure 1A). In contrast to cancer cells, all Wnt antagonist genes were expressed in normal breast tissue (Figure 1A).

### Analysis of Wnt antagonist gene methylation in breast cancer cell lines

We next used MSP to analyse the methylation status of Wnt antagonist genes. A CRC cell line (RKO) in which all of the Wnt antagonist genes were methylated served as a positive control, while another CRC cell line (DKO2) in which the *DNMT1* and *DNMT3B* loci were genetically disrupted served as a negative control (Figure 1B). We observed *SFRP1* methylation in 7 of the 11 (64%) breast cancer cell lines tested (MCF-7, MDA-MB-231, T-47D, SK-BR-3, MDA-MB-361, MDA-MB-453 and ZR-75-1) (Figure 1B). Of those, expression of SFRP1 mRNA was downregulated in five, as described above. We also detected *SFRP1* methylation in two cell lines in which basal expression of the gene remained intact (MDA-MB-361 and ZR-75-1) (Figure 1A and B). As both methylated and unmethylated *SFRP1* was detected in these cells, we suggest that the mRNA was likely transcribed from the unmethylated allele.

*SFRP2* methylation was detected in all 11 cell lines (100%), while *SFRP5* methylation was detected in 10 (91%) (Figure 1B). In general, we observed a good correlation between methylation and the expression status of *SFRP2* and *SFRP5*, although there were some exceptions. For example, some methylation of *SFRP2* was detected in MDA-MB-157 cells (Figure 1B), but they showed a substantial amount of basal mRNA expression (Figure 1A), which was likely transcribed from the unmethylated allele. Conversely, SK-BR-3 cells showed no *SFRP5* methylation but did not express any mRNA. Since DAC treatment did not restore *SFRP5* expression in SK-BR-3 cells, a different mechanism may be responsible for its silencing.

Methylation of *DKK1* was detected in three cell lines (T-47D, MDA-MB-361 and MDA-MB-453) (Figure 1B), although none of the cells showed complete methylation of *DKK1*, and MSP revealed both methylated and unmethylated DNA (Figure 1B). These results are consistent with the observation that two cell lines showed significant downregulation of *DKK1* expression, but the silencing was apparently incomplete, as low levels of expression were detectable with RT-PCR (Figure 1A). In contrast to cancer cell lines, no methylation of Wnt antagonist genes was detected in normal breast tissue (Figure 1B).

**Figure 2:**
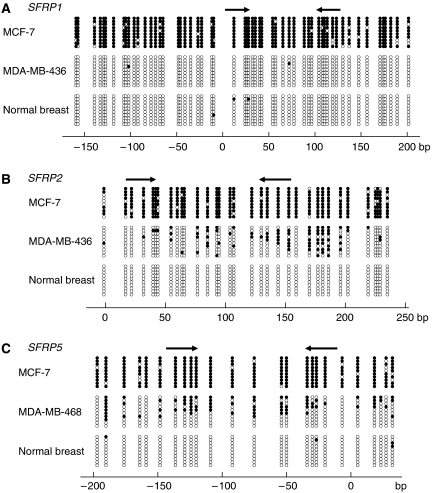
Bisulphite-sequencing analysis of *SFRP* gene methylation. (**A**) Bisulphite sequencing of *SFRP1* in the indicated breast cancer cell lines and normal breast tissue. Open and filled circles represent unmethylated and methylated CpG sites, respectively. The locations of MSP primer sites are shown by arrows on the top. The location of each CpG site relative to the transcription start site is shown below. (**B**, **C**) Bisulphite sequencing of *SFRP2* (**B**) and *SFRP5* (**C**). CpG sites are represented as in panel A.

To analyse the methylation status in more detail, we carried out bisulphite sequencing in selected cell lines. Sequencing analysis revealed that the CpG islands of *SFRP1*, *SFRP2* and *SFRP5* are extensively methylated in MCF-7 cells (Figure 2A–C). By contrast, almost no methylation of *SFRP1* was detected in MDA-MB-436 cells (Figure 2A), which is consistent with the MSP results and the finding that these cells express *SFRP1* (Figure 1). We also analysed *SFRP2* in MDA-MB-436 cells, where both methylated and unmethylated DNA was detected by MSP. Bisulphite sequencing revealed a heterogeneous pattern of *SFRP2* methylation (Figure 2B), and the incomplete silencing of *SFRP2* in this cell line likely reflects the low-density methylation of the gene. A similar heterogeneous methylation pattern was observed for *SFRP5* in MDA-MB-468 cells, where partial methylation was detected with MSP (Figures 1A and 2C). Normal breast tissue showed little or no methylation of *SFRP1*, *SFRP2* or *SFRP5* (Figure 2A–C).

### Analysis of Wnt antagonist gene methylation in primary breast tumours

**Figure 3:**
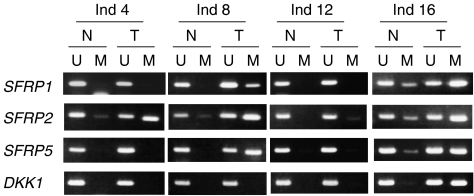
Analysis of methylation of Wnt antagonist genes in primary breast cancers. Shown are representative results of MSP analysis of *SFRP1*, *SFRP2*, *SFRP5* and *DKK1* in primary breast cancer tissues (T) and adjacent normal breast tissues from the same patients (N).

Our finding of epigenetic silencing of *SFRP* genes and *DKK1* in breast cancer cell lines prompted us to determine the extent to which these Wnt antagonist genes are also aberrantly methylated in primary breast tumours. We first analysed *SFRP* methylation in 38 breast tumour specimens and frequently found *SFRP* methylation in these tumours (*SFRP1*, 12 out of 38, 32%; *SFRP2*, 28 out of 38, 74%; *SFRP5*, 22 out of 38, 58%). We then examined the 78 specimens from Japanese breast cancer patients and found the *SFRP* genes to be methylated at similar frequencies (*SFRP1*, 31 out of 78, 40%; *SFRP2*, 60 out of 78, 77%; *SFRP5*, 55 out of 78, 71%). On the other hand, methylation of *DKK1* was detected in only 15 (19%) of the 78 tumours. We also obtained samples of adjacent non-tumourous breast tissue from 20 of the 78 Japanese patients. In general, methylation of *SFRPs* and *DKK1* was tumour-specific or tumour-predominant, but weak methylation of *SFRP* genes was observed in some non-tumourous breast tissues (representative results are shown in Figure 3). This may have been caused by field defects.

The clinicopathological features were available from the Japanese patients, but there were no significant correlations between the methylation status of the Wnt antagonist genes and age, pT status, pN status, pM status, oestrogen receptor status or HER2 status (Table 2).

### SFRPs suppress breast cancer cell growth

**Figure 4:**
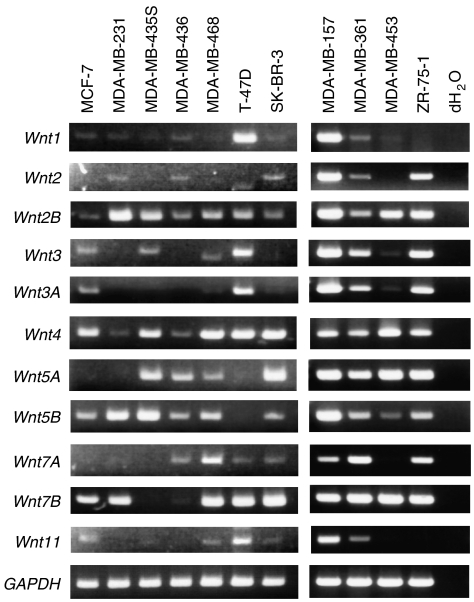
Expression of various Wnt ligands in breast cancer cells. Results of RT-PCR analysis of Wnt ligand gene expression in the indicated breast cancer cell lines are shown. *GAPDH* expression was assessed in all samples to ensure the cDNA quality; dH_2_O indicates no RNA added.

**Figure 5:**
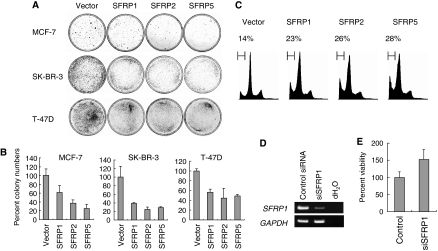
SFRPs suppress breast cancer cell proliferation. (**A**) Representative results from a colony formation assay carried out using the indicated breast cancer cell lines transfected with SFRP or control plasmid (vector). (**B**) Relative colony formation efficiencies of breast cancer cells transfected with SFRP or control plasmid (vector). Shown are means of three replications; error bars represent standard deviations. (**C**) Representative results of a FACS analysis of SK-BR-3 cells transfected with SFRP or control plasmid (vector). Cells were harvested and analysed 48 h after transfection. Apoptotic cells appear as the sub-G1 fraction, and percentages are shown on the top. (**D**) RT-PCR analysis of *SFRP1* expression in MDA-MB-436 cells transfected with control siRNA or siSFRP1. *GAPDH* expression was assessed in all samples to ensure the quality of the cDNA; dH_2_O indicates no RNA added. (**E**) Depletion of SFRP1 upregulated proliferation of breast cancer cells. MDA-MB-436 cells were transfected with control siRNA or siSFRP1, and cell viabilities were determined in MTT assays carried out 48 h after transfection. Values were normalised relative to cells transfected with control siRNA. Shown are means of eight replications; error bars represent standard deviations.

To determine whether *SFRP* genes function as tumour suppressors in breast cancer, we next analysed Wnt signalling in breast cancer cells. We first used RT-PCR to test for the expression of Wnt ligands. We found that all of the cell lines expressed at least 6 of the 11 Wnt ligand genes tested (Figure 4). Subsequent immunofluorescence analysis of the intracellular distribution of *β*-catenin in seven breast cancer cell lines (MCF-7, MDA-MB-231, MDA-MB-435S, MDA-MB-436, MDA-MB-468, T-47D and SK-BR-3) revealed nuclear accumulation of *β*-catenin in four cell lines (MCF-7, MDA-MB-231, T-47D and SK-BR-3) in which *SFRP1*, *SFRP2* and *SFRP5* were silenced (Supplementary Figure 1). No mutation of *APC*, *CTNNB1* or *AXIN* genes has been reported in these cell lines, suggesting that loss of *SFRP* gene function might be responsible for the observed activation of Wnt signalling. To test that idea, we carried out colony formation assays with MCF-7, T-47D and SK-BR-3 cells and found that colony formation was diminished in all cells transfected with any one of the *SFRP* genes (Figure 5A and B). Subsequent FACS analysis confirmed induction of apoptosis in SK-BR-3 cells, showing ectopic expression of SFRPs (Figure 5C).

To test whether the silencing of SFRP expression provides a growth advantage to breast cancer cells, we examined the effect of using siRNA (siSFRP1) to disrupt SFRP1 expression. We initially used three breast cancer cell lines (MDA-MB-435S, MDA-MB-436 and MDA-MB-468) to assess the knockdown efficiency of our siSFRP1, which most effectively disrupted *SFRP1* expression in MDA-MB-436 cells (Figure 5D). Subsequent MTT assays showed that knocking down SFRP1 expression with siSFRP1 increased cell viability, as compared to cells transfected with control siRNA (Figure 5E). Secreted frizzled-related proteins thus appear to suppress breast cancer cell growth, suggesting that they act as tumour suppressors in breast cancer.

### Depletion of SFRP1 upregulates Wnt signalling in breast cancer cells

**Figure 6:**
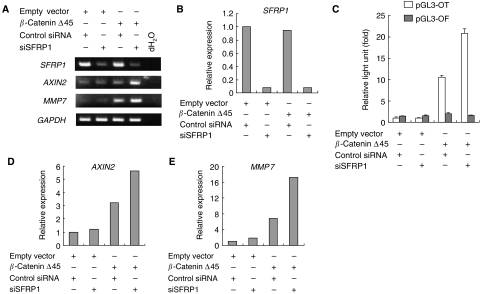
Depletion of SFRP1 upregulates Wnt signalling in breast cancer cells. (**A**) RT-PCR analysis of *SFRP1* and Wnt target genes in MDA-MB-436 cells cotransfected with *β*-cateninΔ45 or an empty vector plus control siRNA or siSFRP1. *GAPDH* expression was assessed in all samples to ensure the cDNA quality; dH_2_O indicates no RNA added. (**B**) Real-time RT-PCR analysis of *SFRP1* in MDA-MB-436 cells cotransfected with *β*-cateninΔ45 or an empty vector plus control siRNA or siSFRP1. Results are normalised to endogenous *GAPDH* expression. (**C**) Relative luciferase activity obtained using a TCF/LEF-responsive reporter (pGL3-OT) or a negative control (pGL3-OF) in MDA-MB-436 cells cotransfected with *β*-cateninΔ45 or an empty vector plus control siRNA or siSFRP1. Results are shown relative to a value of 1 (assigned to cells transfected with empty vector and control siRNA) after correction for transfection efficiency using *Renilla* luciferase activity. Shown are means of four replications; error bars represent standard deviations. (**D**, **E**) Real-time RT-PCR analysis of *AXIN2* (**D**) and *MMP7* (**E**) in MDA-MB-436 cells cotransfected with *β*-cateninΔ45 or an empty vector plus control siRNA or siSFRP1. Results are normalised to endogenous *GAPDH* expression.

To examine the effect of SFRPs on Wnt signalling in breast cancer, we used a TCF/LEF-responsive reporter (pGL3-OT) to analyse the basal TCF/LEF transcriptional activity in seven breast cancer cell lines (MCF-7, MDA-MB-231, MDA-MB-435S, MDA-MB-436, MDA-MB-468, T-47D and SK-BR-3). However, none of the cell lines showed upregulated transcriptional activity (data not shown). We therefore next used siRNA (siSFRP1) to disrupt SFRP1 expression in a breast cancer cell line (MDA-MB-436) and tested whether loss of SFRP function leads to upregulated Wnt signalling. Although transient transfection of siSFRP1 reduced levels of SFRP1 mRNA by approximately 90% (Figure 6A and B), no upregulation in TCF/LEF transcriptional activity was observed (Figure 6C), and expression of TCF/LEF target genes was unchanged (Figure 6A, D and E).

By contrast, transient transfection of a mutant form of *β*-catenin (*β*-cateninΔ45) induced a 10-fold increase of pGL3-OT reporter activity (Figure 6C) and upregulated the mRNA expression of *AXIN2* and *MMP7*, two TCF/LEF target genes (Figure 6A, D and E). Furthermore, reporter assays and analysis of target gene expression revealed that the combination of SFRP1 depletion and *β*-cateninΔ45 overexpression acted synergistically to upregulate TCF/LEF transcription (Figure 6A, C–E).

## DISCUSSION

Compelling evidence suggests that activation of Wnt signalling plays an important role in breast cancer. Immunohistochemical staining carried out by several groups has demonstrated elevated levels of nuclear and/or cytoplasmic *β*-catenin in breast tumours with significant frequency (Lin *et al*, 2000; Ryo *et al*, 2001; Chung *et al*, 2004). Lin *et al* (2000) also showed that nuclear and/or cytoplasmic staining of *β*-catenin correlated with elevated cyclin D1, which is one of the known targets of *β*-catenin/TCF transcription. However, mutation of *APC*, *CTNNB1* or *AXIN* is rare in breast cancer, and thus the mechanism of Wnt signal activation in this disease is not fully understood.

On the basis of their expression array analysis of chromosome 8p11–21 genes, Ugolini *et al* (1999) reported that expression of SFRP1 mRNA is frequently diminished or lost in breast carcinomas, and they also found that loss of *SFRP1* occurs in more than 80% of invasive breast carcinomas, although not in the medullary type (Ugolini *et al*, 2001). Using immunohistochemical analysis, Klopocki *et al* (2004) found that expression of SFRP1 protein was absent in 46% of invasive breast tumours and in 43% of carcinoma *in situ*. In addition, two groups recently reported frequent *SFRP1* methylation in primary breast tumours (Lo *et al*, 2006; Veeck *et al*, 2006), and Bafico *et al* (2004) clearly demonstrated a novel autocrine mechanism leading to constitutive Wnt signalling in breast cancer, which could be suppressed by SFRP1 and DKK1. Taken together, these results suggest that loss of SFRP1 function is a key mechanism by which Wnt signalling is activated in breast cancer.

To date, four *SFRP* family genes (*SFRP1*, *SFRP2*, *SFRP4* and *SFRP5*) and two *DKK* family genes (*DKK1* and *DKK3*) have been identified as targets of epigenetic silencing in human tumours (Suzuki *et al*, 2002; Roman-Gomez *et al*, 2004; Lodygin *et al*, 2005; Aguilera *et al*, 2006; Niehrs, 2006). Among them, we showed that loss of *SFRP1*, *SFRP2* and *SFRP5* contributes to Wnt signal activation in both CRC and GC. Dickkopf proteins bind with LRP5/6 and inhibit Wnt signalling by preventing Wnt and Fz from forming a ternary complex with LRP5/6. However, among the DKK family, DKK3 is the most divergent, as it neither binds LRP5/6 nor significantly affects Wnt singling (Niehrs, 2006). We therefore analysed methylation and expression status of *SFRP1*, *SFRP2*, *SFRP5* and *DKK1*. *SFRP1*, *SFRP2*, *SFRP5* and *DKK1* were methylated in 64, 100, 91 and 27% of the breast cancer cell lines tested, respectively. In primary breast tumours, methylation frequencies of *SFRP1*, *SFRP2*, *SFRP5* and *DKK1* were 40, 77, 71 and 19%, respectively. Although the frequency of *SFRP1* methylation in our samples was somewhat lower than those reported by Lo *et al* (2006) and Veeck *et al* (2006), we also found that a substantial number of breast tumours harbour *SFRP1* methylation. In addition, we found that *SFRP2* and *SFRP5* are methylated in both cultured breast cancer cells and primary breast cancers at quite high frequencies. In contrast to *SFRP* genes, however, the frequency of *DKK1* methylation in breast cancer was relatively limited.

To examine the activity of Wnt signalling in breast cancer, we evaluated the expression status of the Wnt ligands in the cell lines. We previously found that at least 3 of the 11 Wnt ligands were expressed CRC cell lines (Suzuki *et al*, 2004), and even more Wnt ligands were expressed in breast cancer cell lines. Using immunofluorescence analysis, we observed *β*-catenin to be present in the nuclei of four of seven breast cell lines in which multiple *SFRP* genes were methylated and silenced. Ectopic expression of SFRP in breast cancer cells suppressed colony formation and induced apoptosis. Conversely, SFRP1 knockdown enhanced breast cancer cell growth. Thus, loss of Wnt antagonists, particularly SFRPs, appears to contribute to Wnt signal activation in breast cancer.

On the other hand, we found that none of the breast cancer cell lines tested showed upregulated TCF/LEF transcriptional activity, even when they showed a loss of Wnt antagonist genes and nuclear accumulation of *β*-catenin, which is consistent with our earlier findings in GC cells. Although it is well known that mutation in *APC*, *CTNNB1* and *AXIN* is infrequent in GC, we found that the majority of GC cells showed activation and nuclear accumulation of *β*-catenin and methylation of multiple *SFRP* genes (Nojima *et al*, 2007). Among those GC cells, only a few cell lines in which *APC* or *CTNNB1* was mutated showed significant upregulation of TCF/LEF transcriptional activity (Nojima *et al*, 2007). It is thus possible that the TCF/LEF reporter assay used may not be sufficiently sensitive to detect the weak or moderate activation of canonical Wnt signalling.

Alternatively, the tumour-suppressive function of Wnt antagonists may be independent of the Wnt/*β*-catenin pathway. To clarify the functional role of SFRP inactivation in breast cancer, we used siRNA to disrupt endogenous SFRP1 expression. Because none of the breast cancer cell lines presented aberrant TCF/LEF transcription, we used an expression vector encoding a *β*-catenin mutant to boost Wnt signalling. We found that the TCF/LEF transcriptional activity induced by the mutant *β*-catenin was synergistically upregulated when combined with SFRP1 depletion, which is consistent with the recent observation by Fukui *et al* (2005). Using non-small-cell lung carcinoma cells in which *SFRP1* was methylated, they showed that when SFRP1 was cotransfected with mutant *β*-catenin, SFRP1 could attenuate TCF/LEF activity induced by exogenous *β*-catenin. Our results demonstrate the potential of endogenous SFRP1 to inhibit the Wnt/*β*-catenin pathway in breast cancer cells.

In summary, we have shown that epigenetic silencing of multiple Wnt antagonist genes is a common event in breast cancer. In particular, we found that *SFRP2* and *SFRP5* are methylated at even higher frequencies than *SFRP1*. Taken together, our findings indicate that the majority of breast tumours harbour methylation of at least one Wnt antagonist gene, and support the hypothesis that epigenetic silencing of Wnt antagonist genes is a major cause of constitutive Wnt signalling in breast cancer. Our data also suggest that methylation of a set of multiple Wnt antagonist genes may represent a useful marker in breast cancer.

## Figures and Tables

**Table 1:** Sequences for primers and siRNA used in this study

**Gene name**	**Sense**	**Antisense**	**Product size (bp)**
*RT-PCR*			
*SFRP1*	5′-CCAGCGAGTACGACTACGTGAGCTT-3′	5′-CTCAGATTTCAACTCGTTGTCACAGG-3′	497
*SFRP2*	5′-ATGATGATGACAACGACATAATG-3′	5′-ATGCGCTTGAACTCTCTCTGC-3′	322
*SFRP5*	5′-CAGATGTGCTCCAGTGACTTTG-3′	5′-AGAAGAAAGGGTAGTAGAGGGAG-3′	346
*DKK1*	5′-CTTTCTCCCTCTTGAGTCCTTCTG-3′	5′-CATAGCGTGACGCATGCAGCGTT-3′	404
*Wnt1*	5′-GTCTGATACGCCAAAATCCGG-3′	5′-CTCGTTGTTGTGAAGGTTCATG-3′	404
*Wnt2*	5′-TTGAAACAAGAGTGCAAGTGCC-3′	5′-ACTTACACCCACACTTGGTCAT-3′	379
*Wnt2B*	5′-GGACTGATCTTGTCTACTTTGAC-3′	5′-TTGAGTTGAGAGGCTTGAATTGG-3′	338
*Wnt3*	5′-ATGACAGCCTGGCCATCTTTG-3′	5′-AGCCCGTGGCACTTGCATTTG-3′	349
*Wnt3A*	5′-GGCATCAAGATTGGCATCCAG-3′	5′-CACTTGAGGTGCATGTGGCTG-3′	404
*Wnt4*	5′-ATGCTCTGACAACATCGCCTA-3′	5′-TGCGGCTTGAACTGTGCGTTG-3′	333
*Wnt5A*	5′-TGGAAGTGCAATGTCTTCCAAG-3′	5′-AGGTGTTATCCACAGTGCTGCA-3′	314
*Wnt5B*	5′-GAAGCTGTGCCAATTGTACCA-3′	5′-ATCCACAAACTCCTTGGCGAA-3′	355
*Wnt7A*	5′-GCAAGCATCATCTGTAACAAGA-3′	5′-TCTCTTTGTCGCAGCCACAGT-3′	310
*Wnt7B*	5′-CATCAACGAGTGCCAGTACCA-3′	5′-CCTCATTGTTATGCAGGTTCAT-3′	353
*Wnt11*	5′-GAACTGCTCCTCCATTGAGCT-3′	5′-CTTACACTTCATTTCCAGAGAG-3′	364
*AXIN2*	5′-GCCAACGACAGTGAGATATCCAGT-3′	5′-TTGAGGACCCTGGACAGGTGATC-3′	455
*MMP7*	5′-GAATGTTAAACTCCCGCGTCATAGA-3′	5′-CAGCGTTCATCCTCATCGAAGTGA-3′	379
*GAPDH*	5′-CGGAGTCAACGGATTGGTCGTAT-3′	5′-AGCCTTCTCCATGGTGGTGAAGAC-3′	307
			
*Real time RT-PCR*			
*SFRP1*	5′-AGATGCTTAAGTGTGACAAGTTCC-3′	5′-TCAGATTTCAACTCGTTGTCACAG-3′	130
*AXIN2*	5′-TCAAGTGCAAACTTTCGCCAACC-3′	5′-TAGCCAGAACCTATGTGATAAGG-3′	151
*MMP7*	5′-TCACTTCGATGAGGATGAACGC-3′	5′-ATCACTGCATTAGGATCAGAGGA-3′	126
*GAPDH*	5′-CTCTGGTAAAGTGGATATTGTTGC-3′	5′-CCTTGACGGTGCCATGGAATTTG-3′	113
			
*Methylation analysis*			
*SFRP1* MSP-U	5′-GTTTTGTAGTTTTTGGAGTTAGTGTTGTGT-3′	5′-CTCAACCTACAATCAAAAACAACACAAACA-3′	135
*SFRP1* MSP-M	5′-TGTAGTTTTCGGAGTTAGTGTCGCGC-3′	5′-CCTACGATCGAAAACGACGCGAACG-3′	126
*SFRP2* MSP-U	5′-TTTTGGGTTGGAGTTTTTTGGAGTTGTGT-3′	5′-AACCCACTCTCTTCACTAAATACAACTCA-3′	145
*SFRP2* MSP-M	5′-GGGTCGGAGTTTTTCGGAGTTGCGC-3′	5′-CCGCTCTCTTCGCTAAATACGACTCG-3′	138
*SFRP5* MSP-U	5′-GTAAGATTTGGTGTTGGGTGGGATGTTT-3′	5′-AAAACTCCAACCCAAACCTCACCATACA-3′	136
*SFRP5* MSP-M	5′-AAGATTTGGCGTTGGGCGGGACGTTC-3′	5′-ACTCCAACCCGAACCTCGCCGTACG-3′	141
*DKK1* MSP-U	5′-TTAAGGGGTTGGAATGTTTTGGGTTTGT-3′	5′-AAACCTAAATCCCCACAAAACCATACCA-3′	163
*DKK1* MSP-M	5′-AGGGGTCGGAATGTTTCGGGTTCGC-3′	5′-CCTAAATCCCCACGAAACCGTACCG-3′	157
*SFRP1* bis-seq	5′-GTTTTGTTTTTTAAGGGGTGTTGAG-3′	5′-CCAAAAACCTCCGAAAACAAAAAAC-3′	412
*SFRP2* bis-seq	5′-TAAGAAAATTTTGGTTGTGTTTTAGTAA-3′	5′-CAACRAACCAAAACCCTACAACAT-3′	290
*SFRP5* bis-seq	5′-TTAAATGTTTAGGGAGGTAGGGAGT-3′	5′-AATCGCCCAAATAAATAACAACCTAC-3′	293
*DKK1* bis-seq	5′-GCGGGGTGAAGAGTGTTAAAGGTTT-3′	5′-GTCACTTTACAAACCTAAATCCCCAC-3′	277
			
*siRNA*			
*siSFRP1-1*	5′-UCUCUGUGCCAGCGAGUUUtt-3′	5′-AAACUCGCUGGCACAGAGAtt-3′	
*siSFRP1-2*	5′-GCGAGUUUGCACUGAGGAUtt-3′	5′-AUCCUCAGUGCAAACUCGCtt-3′	
*siSFRP1-3*	5′-AGGUGAAGAGCCAGUACUUtt-3′	5′-AAGUACUGGCUCUUCACCUtt-3′	

MSP=methylation-specific PCR; RT=reverse transcriptase; siRNA=small interfering RNA.

**Table 2:** Clinicopathological features of breast cancer with or without Wnt antagonist genes methylation

		***SFRP1***			***SFRP2***			***SFRP5***			***DKK1***		
	***n***	**U**	**M**	***P*-value**	**U**	**M**	***P*-value**	**U**	**M**	***P*-value**	**U**	**M**	***P*-value**
Total	78	47	31		18	60		23	55		63	15	
Age (years, mean±s.d.)		53.66±11.01	52.22±11.97		53.56±12.03	52.95±11.24		52.78±8.88	53.22±12.31		52.46±11.43	55.73±10.96	
													
*pT category*													
pTis	1	0	1	0.796	0	1	0.629	0	1	0.860	1	0	0.129
pT1	17	12	5		4	13		6	11		13	4	
pT2	41	25	16		11	30		10	31		32	9	
pT3	8	4	4		1	7		5	3		8	0	
pT4	7	5	2		1	6		1	6		7	0	
													
*pN category*													
pN0	35	20	15	0.809	9	26	0.678	7	28	0.125	29	6	0.747
pN1	28	20	8		6	22		11	17		21	7	
pN2	6	2	4		2	4		1	5		6	0	
pN3	3	2	1		0	3		2	1		3	0	
													
*pM category*													
pM0	66	40	26	1.000	17	49	0.325	20	46	0.664	53	13	0.583
pM1	6	4	2		0	6		1	5		6	0	
													
*ER*													
Negative	15	10	5	0.517	6	9	0.292	6	9	0.128	14	1	0.286
Positive	26	13	13		5	21		4	22		19	7	
													
*HER2*													
Negative	24	15	9	0.353	9	15	0.086	5	19	0.714	19	5	0.714
Positive	18	8	10		2	16		5	13		15	3	
													
*Histology*													
Ductal carcinoma *in situ*	4	3	1		2	2		1	3		4	0	
Invasive ductal carcinoma	63	37	26		11	52		18	45		51	12	
Lobular carcinoma *in situ*	0	0	0		0	0		0	0		0	0	
Invasive lobular carcinoma	2	1	1		0	2		1	1		1	1	
Others	7	6	1		4	3		3	4		5	2	

ER=oestrogen receptor; HER2=human epidermal growth factor receptor 2.
