# Frequent epigenetic inactivation of Wnt antagonist genes in breast cancer

**DOI:** 10.1038/sj.bjc.6604507

**Published:** 2008-07-15

**Authors:** H Suzuki, M Toyota, H Carraway, E Gabrielson, T Ohmura, T Fujikane, N Nishikawa, Y Sogabe, M Nojima, T Sonoda, M Mori, K Hirata, K Imai, Y Shinomura, S B Baylin, T Tokino

**Correction to**: *British Journal of Cancer* (2008) **98**, 1147–1156; doi:10.1038/sj.bjc.6604259

Owing to an error on the part of the authors, the third author of this paper, H Carraway, was originally submitted and subsequently published as ‘H Caraway’. The correct spelling is given in the author list above.

