# Supplementary material for: Frequent epigenetic inactivation of Wnt antagonist genes in breast cancer
Source: Br J Cancer. 2008 Feb 19;98(6):1147–56. doi: 10.1038/sj.bjc.6604259 (PMC2275475; doi:10.1038/sj.bjc.6604259)
Supplement: Supplementary Figure 1 [file 6604259x1.ppt]

## Slide 1
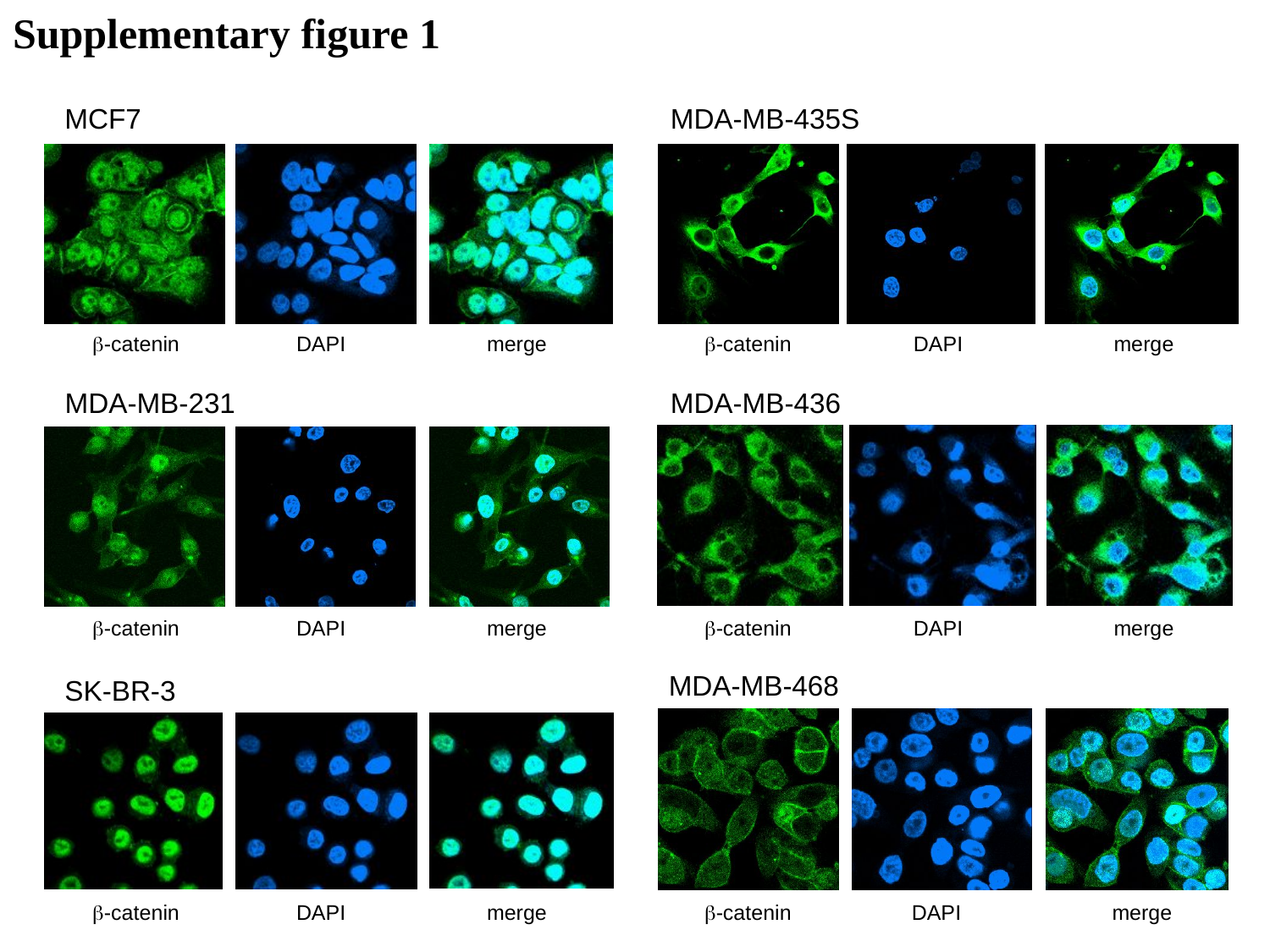

Supplementary figure 1
MCF7
MDA-MB-435S
β-catenin
DAPI
merge
β-catenin
DAPI
merge
MDA-MB-231
MDA-MB-436
β-catenin
DAPI
merge
β-catenin
DAPI
merge
MDA-MB-468
SK-BR-3
β-catenin
DAPI
merge
β-catenin
DAPI
merge
